# Insights into the Bioinformatics and Transcriptional Analysis of the Elongator Complexes (*ELPs*) Gene Family of Wheat: *TaELPs* Contribute to Wheat Abiotic Stress Tolerance and Leaf Senescence

**DOI:** 10.3390/plants12040952

**Published:** 2023-02-20

**Authors:** Feng Guo, Md Ashraful Islam, Chenxu Lv, Xiujuan Jin, Lili Sun, Kai Zhao, Juan Lu, Rongyue Yan, Wenjun Zhang, Yugang Shi, Ning Li, Daizhen Sun

**Affiliations:** 1 State Key Laboratory of Sustainable Dryland Agriculture, College of Agronomy, Shanxi Agricultural University, Taigu 030801, China; 2 Department of Biological Sciences, University of North Texas, Denton, TX 76201, USA

**Keywords:** Elongator complexes (*ELPs*), in silico analysis, abiotic stress, leaf senescence, wheat

## Abstract

Elongator complexes (*ELPs*) are the protein complexes that promote transcription through histone acetylation in eukaryotic cells and interact with elongating RNA polymerase II (RNAPII). *ELPs*’ role in plant growth and development, signal transduction, and response to biotic and abiotic stresses have been confirmed in model plants. However, the functions of the wheat *ELP* genes are not well documented. The present study identified 18 members of the *ELPs* from the wheat genome with a homology search. Further, bioinformatics and transcription patterns in response to different stress conditions were analyzed to dissect their potential regulatory mechanisms in wheat. Gene duplication analysis showed that 18 pairs of *ELP* paralogous genes were derived from segmental duplication, which was divided into six clades by protein phylogenetic and cluster analysis. The orthologous analysis of wheat *TaELP* genes showed that *TaELP* genes may have evolved from orthologous genes of other plant species or closely related plants. Moreover, a variety of cis-acting regulatory elements (CAREs) related to growth and development, hormone response, and biotic and abiotic stresses were identified in the *TaELPs’* promoter regions. The qRT-PCR analysis showed that the transcription of *TaELPs* was induced under hormone, salt, and drought stress and during leaf senescence. The *TaELP2* gene was silenced with BSMV-VIGS, and *TaELP2* was preliminarily verified to be involved in the regulation of wheat leaf senescence. Overall, *TaELP* genes might be regulated by hormone signaling pathways and response to abiotic stress and leaf senescence, which could be investigated further as potential candidate genes for wheat abiotic stress tolerance and yield improvement.

## 1. Introduction

RNA polymerase II (RNAPII)-related factors can affect both transcription initiation and elongation phases. Although promoter-regulated transcription initiation requires the binding of transcription factors, and the multisubunit Srb/mediator complex-bound RNAPII holoenzyme plays an important role in promoters in Saccharomyces cerevisiae, RNAPII can prolong transcripts without transcription factors [1,2]. Otero et al. [3] found that the Elongator complexes (*ELPs*) were the main component of the extended C-terminal repeat domain (CTD)-highly phosphorylated RNAPII holoenzyme, which affected the elongation of RNAPII transcripts through effects on chromatin under specific conditions. *ELP* is a protein complex composed of six subunits (ELP1–ELP6), of which ELP1–ELP3 subunits formed the core complex, and ELP4–ELP6 subunits were auxiliary complexes [4]. In *S. cerevisiae*, the six genes encoding the *ELP* subunits are all indispensable for yeast growth and affect sensitivity to high salt, caffeine, and 6-azouracil phenotypes, suggesting that six subunits are essential elements for studying the elongation factor complex function [5,6,7]. ELP1 (also known as IKI3, IKBKAP, and IKAP), the largest subunit of Elongator (~150 kDa), is highly conserved in eukaryotes and involved in pro-inflammatory cytokine signaling as a regulator of IκB kinases [8]. It contains a conserved WD40 domain, a C-terminal basic region, and a phosphorylated segment and is involved in promoting tRNA binding and modification [9,10]. ELP2 (~90 kDa) was the second-largest subunit compared to the other six subunits; it contains two WD40 domains arranged in tandem and can link ELP1 and ELP3 [5,9]. ELP3 is considered to be the catalytic subunit of the Elongator complex, which is the enzymatic core protein of *ELP* and contains an N-terminal iron-sulfur (Fe-S) radical SAM (S-adenosylmethionine) domain and a C-terminal GNAT-type histone acetyltransferase (HAT) domain [9,11,12]. In the Elongator complex, ELP1, ELP2, and ELP3 form the core complex; ELP4, ELP5, and ELP6 form the accessory complex; and they assemble to form a RecA-type ATPase with a hexameric ring structure and are involved in tRNA binding and uridine modification at swing position [7,9,13,14]. However, *ELP* is involved in various cellular gene regulations and biological signal transduction, such as tRNA modification, histone modification, DNA demethylation or methylation, tubulin acetylation, exocytosis, etc. [15,16].

In the natural environment, the growth and development of plants suffer from biotic and abiotic adversities all the time. To avoid these stresses, plants develop various immune defense mechanisms [17]. When plants are subjected to external environmental hazards, cells have been instructed to switch from normal growth and development to stress response. After the stress response is completed, the physiological state is restored to normal growth and development. The transformation of these physiological states is inseparable from the reprogramming of transcriptome, and the strength of plant transformation ability is related to the degree of change in initiating the reprogramming of transcriptome, in which *ELP* complex plays an important regulatory role [18,19]. Nelissen and Berná et al. [20,21,22,23] found that compared to the control, *Arabidopsis elo3 (Atelp3)* mutants had lower germination and seed setting rates at the seedling stage, slower germination, delayed flowering, abnormal growth of shoot morphological structures of leaves and flowers, reduced leaf area, irregularly developing apical meristem, and slow growing underground primary roots and hypocotyls. Xiaofeng et al. [19] isolated four Elongator-related mutants from *Arabidopsis* ABA-sensitive mutants, and the observed phenotypes showed slow leaf and root growth, poor development, and increased ABA sensitivity and anthocyanin accumulation. Furthermore, Zhizhong et al. [24] screened mutants related to drought stress, and they found that *Arabidopsis AtELP1* mutants were highly sensitive to ABA during growth and development and accelerated stomatal closure to battle drought stress, indicating that *AtELP1* plays an important role in ABA signal transduction. Disruption of the Elongator complex in *Arabidopsis* caused altered plant physiological signaling and enhanced resistance to oxidative stress induced by CsCl and methyl viologen, suggesting that *ELP* plays a negative regulatory role in the oxidative stress response [19]. In *Arabidopsis* pathogen sensing, *AtELP2* was found to induce defense gene expression and initiate effector-triggered immunity against pathogen invasion [25]. In the screening of *Arabidopsis Atelp2* mutants, mutant *Atelp3* was found to respond to pathogens, similarly to *AtELP2*, but did not initiate systemic acquired resistance (SAR) defense mechanisms [26]. It is speculated that when plants encounter pathogen invasion, basal immunity and ETI defense mechanisms are induced by the Elongator complex, but arebnot related to SAR, and the Elongator complex may induce the transcription of defense genes through the effect on chromatin [26]. Heterologous expression of *Arabidopsis AtELP3* and *AtELP4* in tomatoes can significantly increase the resistance of transgenic tomatoes to *Pseudomonas syringae* without affecting plant growth and development [27]. In wheat, silencing of *TaELP4* reduces resistance to *Bacillus cereus* and significantly reduces the expression of related defense genes such as *TaAGC1, TaCPK7-D,* and *TaPAL5* and inhibits chitinase 2 histone acetylation levels [28]. Thus, the above studies have shown that *ELP* plays an important role in plant growth and development, biotic stress, and abiotic stress, and the same *ELP* subunit may participate in different signaling pathways by interacting with multiple downstream targets and mediate plant resistance to multiple stresses.

However, the research on plant *ELP* is mainly concentrated in the model crop *Arabidopsis*, and the study on other crops’ *ELP* is relatively less. Wheat is grown all over the world and provides an indispensable caloric resource for human beings [29]. As world population grows, wheat production is projected to increase by 38% to meet the growing food demand [30]. However, little is known about the *ELP* gene family in wheat. Compared with the genomes of other crop plants, bread wheat is heterologous, has the largest genome (16 Gb genome size; AABBDD genome), and contains a large number of repeats and transposable elements, which has caused great difficulties in identifying good candidate genes and breeding of wheat [31,32]. With the widespread application of genome sequencing in plants, wheat whole genome sequencing and annotation are also published online [33]. Wheat genome sequencing data in combination with the in silico approach lays the foundation for us to identify and annotate the *ELP* gene family in wheat and facilitates the exploration of potential candidate genes of the *ELP* gene family [34]. In this study, we used bioinformatics analysis to predict and analyze the number of *TaELP* genes based on the whole genome of wheat, and gene structure, conserved domains, cis-acting elements, evolutionary relationships, subcellular localization, protein–protein interaction prediction, hormones, and abiotic stress-induced expression patterns were analyzed. Our study will provide a theoretical basis for further research in the biological function of the wheat *TaELP* gene family members under abiotic stress.

## 2. Results

### 2.1. Identification and Annotation of Wheat ELP Family Members

*Arabidopsis* and rice *ELP* proteins were used as reference sequences to search in the wheat genome database. After HMM and smart analysis, 18 wheat *ELP* genes (Table 1 and Table S1) were finally identified and named according to their physical positions on the chromosome. The members of the wheat *ELP* family were further annotated by gene ID, position, open reading frame (ORF) length, and protein physicochemical properties. The ORFs of *TaELPs* ranged from 753 to 3978 bp, and the protein length ranged from 250 to 1325 amino acids. The molecular weights of *TaELPs* ranged from 27.05 to 147.31 KDa, and according to the predicted isoelectric point (PI) values, the PI ranged from 5.30 to 8.97, of which six genes were found to be basic (>7) and 12 genes were found to be acidic (<7) [35].

The aliphatic amino acid index and instability index were calculated. The aliphatic amino acid index ranged from 79.35 to 101.83, and the instability index ranged from 31.37 to 53.49. The high aliphatic amino acid index of the protein sequence indicates that it can play a role in a wide temperature range, while the instability index indicates whether the protein is stable or unstable [35]. Among them, six genes were stable (instability index < 40), and the remaining *TaELPs* were unstable (instability index > 40) [36]. The calculated hydropathic index (GRAVY) of *TaELPs* ranges from −0.085 to 0.193, indicating that they were hydrophilic and can better interact with water [36]. WoLF PSORT (https://wolfpsort.hgc.jp/, accessed on 20 July 2021) and CELLO v.2.5(http://cello.life.nctu.edu.tw, accessed on 20 July 2021) were used to predict subcellular localization. Subcellular localization prediction of *TaELP* showed that most *TaELP* family members were localized in the cytoplasm. Furthermore, 3 TaELPs (TaELP6-A, TaELP6-B, TaELP6-D) were localized in the plastid, and TaELP2-A, TaELP2-B, and TaELP2-D were in the cytoplasm, nucleus, and plasma membrane, respectively.

### 2.2. Chromosomal Distribution and Gene Duplication of TaELPs

**Figure 1:**
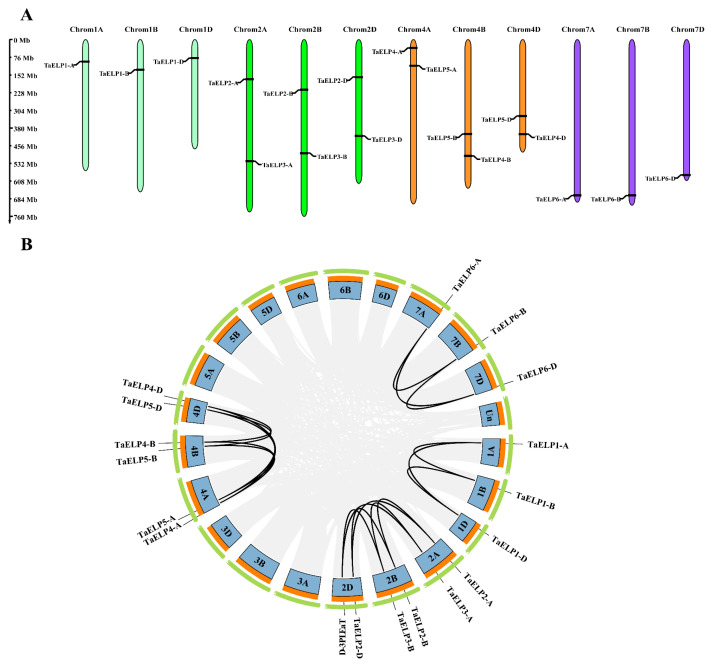
(**A**) Illustration of the chromosomal distribution of *TaELPs* on wheat chromosomes. The gene name is around the chromosome, and the chromosome numbers of three subgenomes are displayed at the top of each chromosome. (**B**) Genomic localization and replication event analysis of 18 *TaELPs* genes. Light gray lines in the background indicate syntenic blocks within the bread wheat genome. Duplicate events are highlighted with black lines.

Eighteen *TaELP* genes of wheat were located on 12 wheat chromosomes (Figure 1 and Table 1). *TaELPs* genes were evenly distributed in A, B, and D subgenomes, and each subgenome contained six *TaELP* genes (Figure 1). Chromosomes 2A, 2B, 2D, 4A, 4B, and 4D comprised two genes, while 1A, 1B, 1D, 7A, 7B, and 7D contained one gene each (Figure 1). No *TaELP* genes were found on chromosomes 3, 5, and 6, suggesting that *TaELP* family genes were unevenly distributed throughout the wheat chromosomes.

Gene duplication analysis showed that there were 18 pairs of *ELP* paralogous genes in the wheat genome (Figure 1 and Table S2), all of which were derived from segmental duplication and located at conserved positions in segmental duplication regions on different chromosomes, indicating that segmental duplication played an important role in the quantitative expansion of wheat *ELP* genes [37]. Two segmental duplications occurred on chromosomes 2A, 2B, 2D, 4A, 4B, and 4D, and one segmental duplication occurred on chromosomes 1A, 1B, 1D, 7A, 7B, and 7D (Figure 1 and Table S2). We used TBtools software to calculate the nonsynonymous mutation rate (Ka), synonymous mutation rate (Ks), and the ratio of nonsynonymous mutation rate (Ka) to synonymous mutation rate (Ks) (Ka/Ks) (Table S2). The value Ka/Ks = 1 denotes that genes experienced a neutral selection, <1 suggests a purifying or negative selection, and >1 indicates a positive selection [38]. The Ka/Ks values of the 18 pairs of *ELP* paralogous genes were all less than 1, suggesting that the *TaELP* genes all go through purification selection after fragment duplication, and the divergence time ranged from 1.83 to 8.53 million years ago (MYA). In conclusion, these results indicate the conserved evolution of *TaELP* genes in wheat.

### 2.3. Phylogenetic and Cluster Analysis of Wheat TaELPs

**Figure 2:**
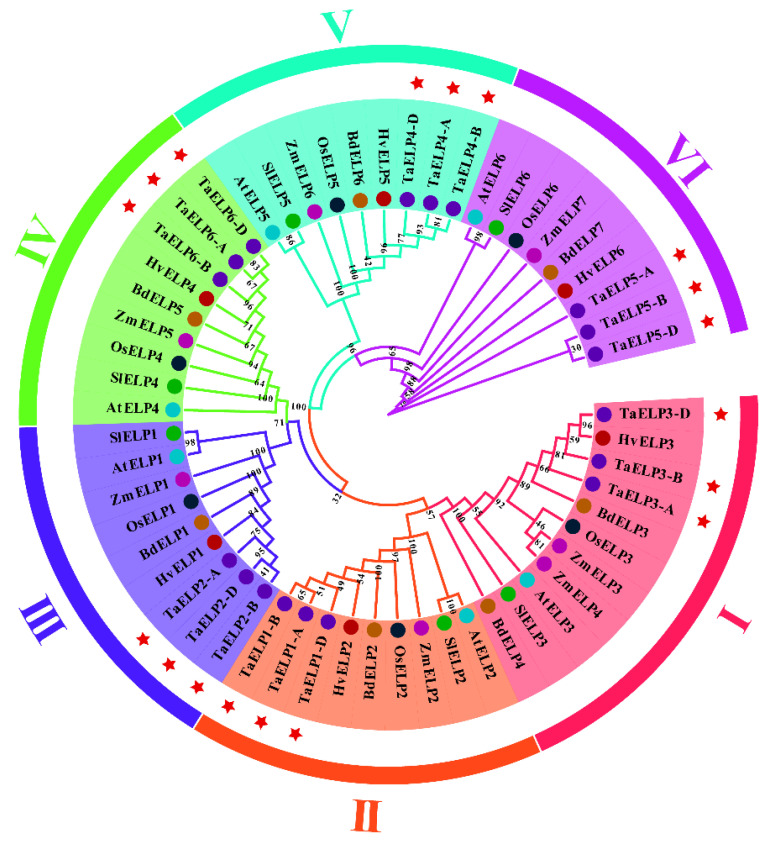
Phylogenetic analysis of TaELP proteins. The tree was generated using MEGA X with the neighbor-joining (NJ) method with 1000 bootstrap values. *TaELPs* are represented by red five-pointed stars. The abbreviations represent the species as follows: Ta, *Triticum aestivum*; Os, *Oryza sativa*; Zm, *Zea mays*; At, *Arabidopsis thaliana*; Hv, *Hordeum vulgare*; Bd, *Brachypodium distachyon*; Sl, *Solanum lycopersicum*. All the species and protein ID used for constructing the tree are presented in Table S3.

To further understand the evolutionary relationship and phylogeny of *TaELPs* and *ELPs* from other plant species, we constructed a phylogenetic tree of *ELP* protein sequences from seven plant species (Figure 2 and Table S3) by neighbor-joining (NJ). Phylogenetic tree results indicated (Figure 2) that *ELP* proteins were divided into six clades. Among them, clade I was the largest, containing 11 members. Clade II to Clade VI each contained nine members. Each clade contained both monocotyledonous and dicotyledonous ELP proteins, indicating that the structural features of *ELP* proteins evolved before the separation of monocotyledonous and dicotyledonous plants. Wheat *ELP* proteins were closely related to *Zea mays, Hordeum vulgare, Brachypodium distachyon*, and *Oryza sativa*, indicating that *ELPs* from these species were highly conserved in protein sequences and could have similar functions.

The 18 wheat *ELP* proteins were evenly divided into six clades, each of which contained A, B, and D subgenomes. We compared the protein sequence similarity of the A, B, and D subgenomes of the same group, and the results showed that the similarity was more than 95% (Table S4). Studies have shown that the protein sequence similarity and identity of gene duplications exceed 70% and 75%, respectively [39]. By analyzing the protein sequence and the constructed phylogenetic tree (Figure 2 and Table S4), it was further confirmed that there is a gene duplication event in the wheat *TaELP* family genes.

### 2.4. Orthologous Analysis of Wheat TaELPs Genes

**Figure 3:**
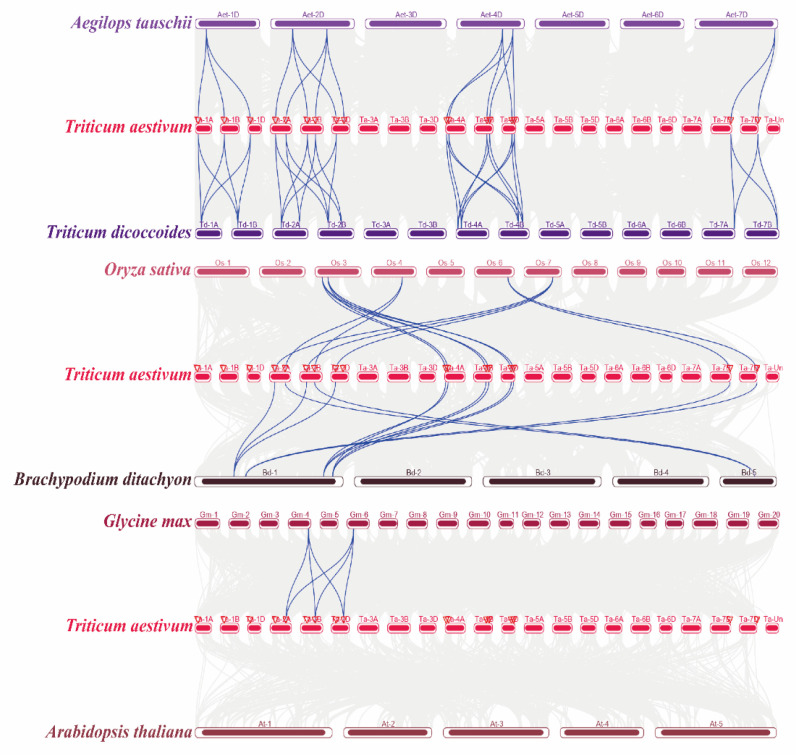
The syntenic relationship between *ELPs* of wheat and *Oryza sativa, Arabidopsis, Brachypodium, Glycine max, Triticum dicoccoides*, and *Aegilops tauschii*. Collinear blocks in wheat and other plant genomes are represented by gray lines in the background, while syntenic *TaELP* gene pairs are highlighted with blue lines.

To study the evolutionary relationship of the wheat *TaELP* gene family, McscanX software was used to visualize the results of collinearity analysis. We selected the dicotyledonous plants (*Arabidopsis* and *Glycine max*), monocotyledonous plants (*Oryza sativa*), and wheat relatives (*Brachypodium distachyon*, *Triticum dicoccoides*, and *Aegilops tauschii*) to identify orthologous gene pairs of the wheat *ELP* genes (Figure 3 and Table S2). We identified a total of 56 orthologous gene pairs of *ELP* genes (Table S2). No *ELP* orthologous gene pairs were observed between *Arabidopsis* and wheat (At-Ta), and only three *ELP* orthologous gene pairs were found between *Glycine max* and wheat (Gm-Ta). Thirteen *ELP* orthologous gene pairs were found between *O.sativa* and wheat (Os-Ta). We also found that there are 13, 10, and 17 orthologs of *ELPs* genes between wheat relatives *Brachypodium distachyon*, *Triticum dicoccoides*, and *Aegilops tauschii* (Bd-Ta, Td-Ta, and Aet-Ta) and wheat. These results suggest that *ELP* genes in wheat are distantly related to those in dicotyledonous species and are closely associated with those in *Aegilops tauschii*, which might be because *Aegilops tauschii* are widely considered to be the D-genome ancestor of wheat [32,40]. The Ka/Ks ratio indicates the selection pressure of plant genes and can be used to diagnose the evolutionary form of the sequence [41]. Thus, we calculated the Ka, Ks, Ka/Ks, and T values of all orthologous gene pairs in wheat to further investigate the evolutionary trends of the *ELP* gene family (Table S2). The results showed that the Ka/Ks ratios of all orthologous genes (Ta-Gm, Ta-Os, Ta-Bd, Ta-Td, and Ta-Aet) were less than 1, suggesting that purification selection plays a dominant role in the evolutionary trend of the *ELP* gene family. According to the divergence time T value calculated from the Ks value, we found that the divergence times of the orthologous genes (Ta-Gm, Ta-Os, Ta-Bd, Ta-Td, and Ta-Aet) were different, among which the orthologous genes (Ta-Gm) had the longest divergence time and had the shortest divergence time with *Aegilops tauschii*. In conclusion, *TaELP* genes in wheat may have evolved from orthologous genes of other plant species or closely related plants.

### 2.5. Gene Structure and Conserved Motif Analysis of TaELPs Genes

**Figure 4:**
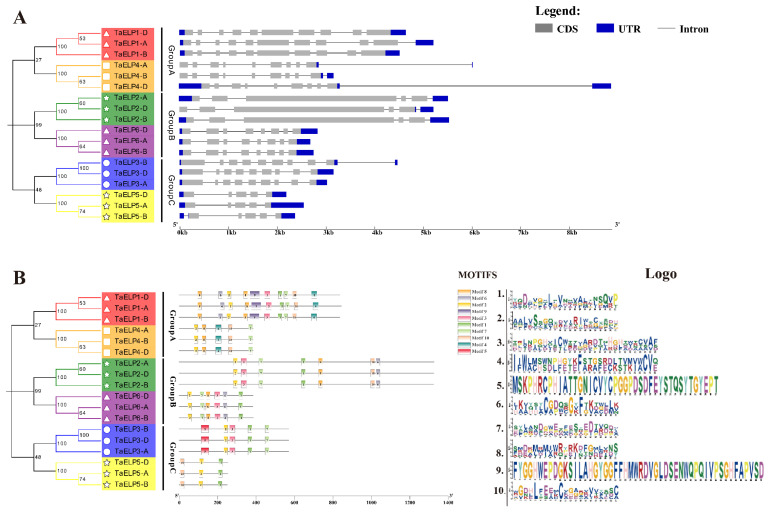
Gene structure and motif analysis of *TaELPs*. (**A**) Introns are represented with black lines, exons are represented with gray boxes, and untranslated regions (UTRs) are represented with blue boxes. (**B**) Conserved motifs of members of *TaELPs*. Ten patterns were identified using the MEME program and are presented with boxes of different colors. Legend: Represents gene structure. Logo: Indicates different MOTIFS patterns.

The phylogenetic tree constructed based on the protein sequences of the members of the wheat *TaELP* gene family showed that the members of the *TaELP* gene family were divided into three groups (Group A, Group B, and Group C), and the results were visualized by combining the gene structure and conserved motifs (Figure 4). The gene structure analysis of wheat *TaELPs* found that introns ranged from 4 to10, and exons ranged from 5 to11. A maximum of 11 exons were found in *TaELP1-A*, while a minimum of five introns were found in *TaELP5-A* and *TaELP5-D* (Figure 4A). The exon–intron numbers and structure of most genes were relatively conserved in the *TaELP* members of Group A, Group B, and Group C.

To further understand the structural diversity of wheat *ELPs*, we submitted the protein sequences of 18 *TaELP* genes to the MEME5.4.1 online website and predicted 10 conserved motifs (Figure 4B). The results showed that the number of conserved motifs ranged from three to nine. All *TaELP* gene family members contained Motif 2, while *TaELP4-A*, *TaELP4-B*, and *TaELP4-D* lack Motif 1. Motif 5 was unique to *TaELP3-A*, *TaELP3-B*, and *TaELP3-D*. The same group of ELP proteins contained similar motifs, and they may also have similarities in gene function. For example, Motif 2, Motif 8, Motif 4, Motif 10, and Motif 7 were included in Group A. The differences in the types and numbers of conserved motifs in wheat *ELP* proteins reflected the structural diversity of these proteins, indicating that they might have different biological functions.

### 2.6. Protein Conservation Domain and 3-D Protein Structure Analysis of TaELPs Gene

**Figure 5:**
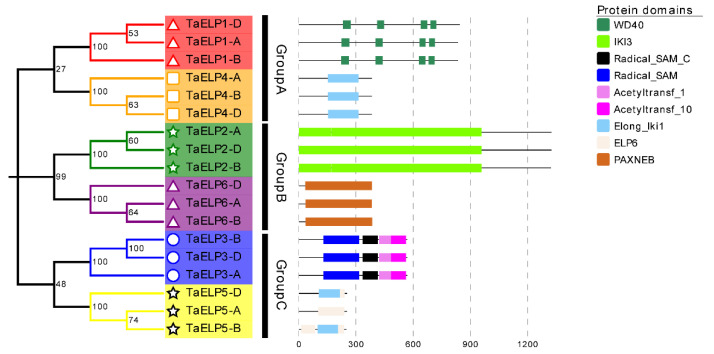
The conserved domain of *TaELP* members was identified from Pfam and SMART databases and presented using Evolview.

The Pfam database was utilized to find the conserved domains of *TaELPs* proteins [42]. The conserved domains of *TaELPs* are shown in Figure 5. TaELP1-A, TaELP1-B, and TaELP1-D contain four WD40 (WD domain, G-beta repeat) protein domains; TaELP4-A, TaELP4-B, and TaELP4-D contain one Elong_Iki1 (Elongator subunit Iki1) domain; TaELP2-A, TaELP2-B, and TaELP2-D contain 1 IKI3 domain; TaELP6-A, TaELP6-B, and TaELP6-D contain 1 PAXNEB domain; TaELP3-A, TaELP3-B, and TaELP3-D contain a catalytic domain of S-adenosylmethionine (Radical SAM superfamily) and a histone acetyltransferase (Acetyltransferase (GNAT) family) domain; and TaELP5-A, TaELP5-B, and TaELP5-D all contain an ELP6 (Elongator complex 6) domain. In addition, an Elong_Iki1 (Elongator subunit Iki1) domain was found in TaELP5-B and TaELP5-D.

We used SWISS-MODEL to further identify 3D models of TaELPs proteins [35,43], and the 3D structure reveals a few key residues linked to biological processes or intended outcomes (Figure S1). For TaELP1-A, TaELP1-B, and TaELP1-D proteins, 3D structures were analyzed using the template “6qk7.1.B”, a template that describes Elongator complex protein 2. For TaELP2-A, TaELP2-B, and TaELP2-D proteins, 3D structures were analyzed using the “6qk7.1.A” template, a template describing the Elongator complex protein 1.; the 3D structure was analyzed using the “6qk7.1.C” template for TaELP3-A, TaELP3-B, and TaELP3-D protein, a description of Elongator complex protein 3, and wasbidentified as containing two ligands (1 × 5AD and 1 × SF4), of which, 5AD (5’-DEOXYADENOSINE) 9 residues were found within 4Å and 4 PLIP interactions, and SF4 (IRON/SULFUR CLUSTER) 8 residues were found within 4Å and 3 PLIP interactions. TaELP4-A, TaELP4-B, and TaELP4-D 3D structures were analyzed using the ”4a8j.1.B” template; the “4a8j.1.B” template is a description ELONGATOR COMPLEX PROTEIN 5. For TaELP5-A, the “4ejs.1.C” template was used to analyze 3D structure, and for TaELP5-B and TaELP5-D proteins, “4wia.1.A” was used to analyze 3D structure. For TaELP6-A, TaELP6-B, and for TaELP6-D proteins, the “4a8j.1.A” template was used to analyze 3D structures. The residues in the favored region of the Ramachandran plots generated by all *TaELPs* ranged from 86.39% to 94.32, the residues in the outlier region ranged from 0.44% to 6.27%, and the coverage of most *TaELPs* was above 80%. Only TaELP4-A, TaELP4- B, and TaELP4-D protein coverage was 58% (Table S5).

In addition, we also used SOPMA to calculate the secondary structure elements of the protein sequence (Table S6); the results showed that the *TaELP’s* protein α-helix (Alpha helix) ranged from 13.52% to 44.46%, β-turn (Beta turn) ranged from 3.39% to 10.24%, the random coil ranged from 30.31% to 49.94%, and the extended strand ranged from 9.66% to 32.30%.

### 2.7. Cis-Acting Element Regulation (CARE) Analysis of Wheat TaELPs Genes

**Figure 6:**
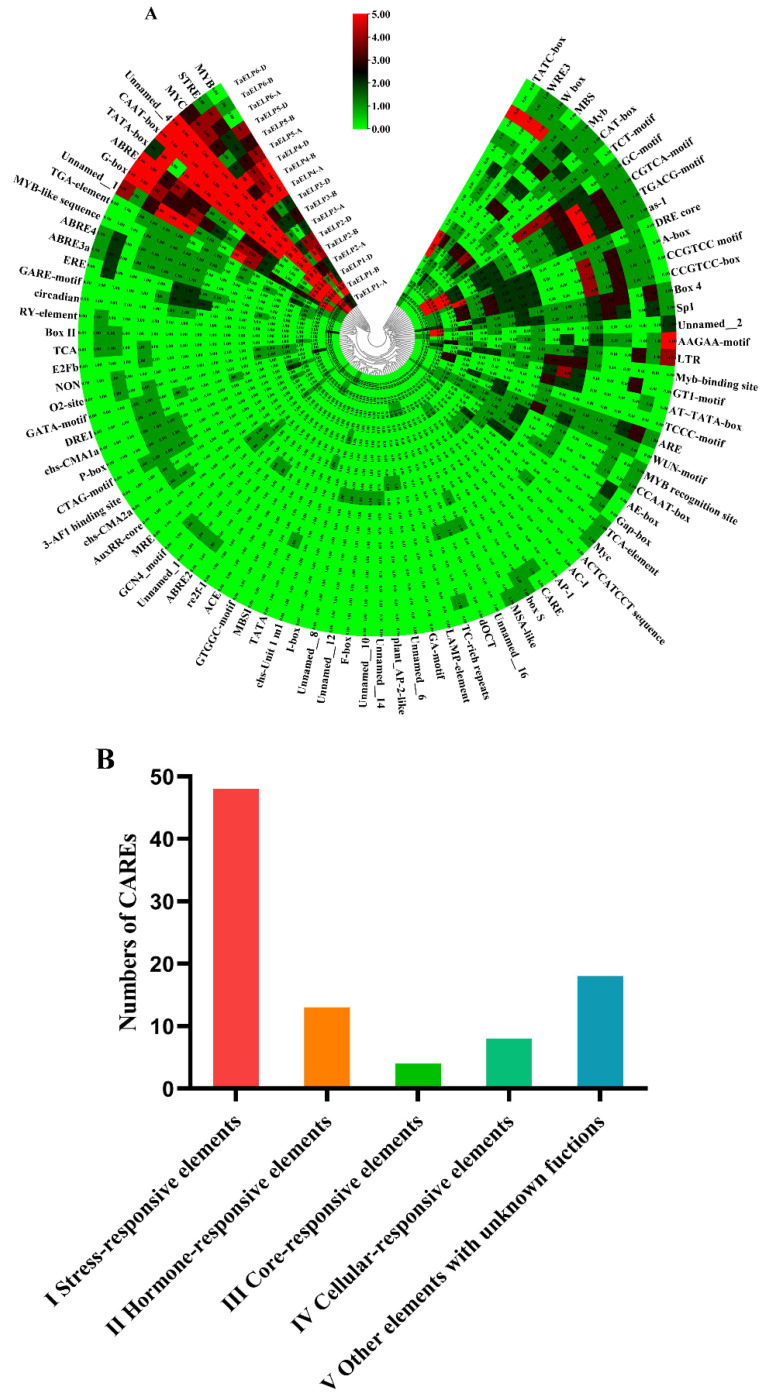
Cis-regulatory elements (CAREs) of the *TaELP* gene family. CAREs analysis of the 2 kb upstream region was performed using the PlantCARE online server. (**A**) Red indicates CREs with higher frequencies, while green indicates CREs with zero frequencies. (**B**) Grouping of CAREs in the *TaELP* gene family.

To examine the responses of *TaELP* members to various environmental stimuli, the 2 kb promoter sequences upstream of the start codon of these genes were submitted to the PlantCARE service to predict their CAREs. A total of 91 different CAREs were identified by analyzing the upstream 2000 bp promoter region of the *TaELP* gene, mainly investigating abiotic stress and defense-related hormone response elements. All the identified CAREs were divided into five groups according to their known functions (Figure 6 and Table S7). Group I contained 48 environmental stress-related CAREs. Among them were 14 different types of abiotic stress response elements, one cis-element involved in low-temperature response (LTR), one cis-acting element (DRE core) regulating cold and dehydration response gene expression, three anaerobic-induced essential cis-regulatory elements (ARE, GC-motif, plant_AP-2-like), one MYB binding site (MBS) associated with drought induction, and eight stress-response-related response elements (such as MYC, as-1, Unnamed__1, WRE3, etc.) (Figure 6A and Tables S7 and S8). The four cis-acting elements were related to wounding and pathogen response (box S, TC-rich repeats, W box, CCAAT-box), and the rest were light-responsive elements of different types, such as 3-AF1 binding site, AE-box, Box II, GT1-motif, chs-CMA1a, etc.

Group II contained hormone response-related CAREs. There was a total of 13 different types of CAREs that regulate hormone response, such as cis-acting elements involved in abscisic acid response (ABRE, ABRE2), cis-regulatory elements involved in MeJA response (CGGTA-motif, TGACG-motif), cis-regulatory elements (AuxRR-core, TGA-element) involved in auxin response, cis-acting elements (TCA-element, TGACG-motif) related to salicylic acid response, etc. Group III contained four core cis-acting elements, among which CAAT-box and TATA-box appear most frequently in all *TaELP* genes, indicating that they play an important role in transcription initiation. TATA-box (including TATA and ATTATA-box) and CAAT-box cis-elements are promoter-associated elements that function at the initiation of transcription [35]. Group IV was plant growth and development-related CAREs, including cis-elements involved in seed-specific expression (AAGAA-motif, RY-element), cis-acting elements involved in cell cycle regulation (MSA-like), meristem expression associated cis-regulatory elements (CAT-box), and several other CAREs associated with cell division. Group V was a small number of CAREs with unknown functions, which are also commonly found in the promoter sequences of *TaELP* genes, indicating that they may also be involved in the regulatory mechanism of *TaELP* genes in the environment (Figure 6 and Table S7).

In conclusion, *TaELP* genes may be involved in the regulation of the above environmental stress-related, phytohormone responses and cell growth and development. 

### 2.8. TaELP Expression Pattern Prediction Analysis

To further explore the expression patterns of *TaELP* genes in different tissues, developmental stages, and abiotic stresses in wheat, we retrieved all wheat mRNA transcription data from the wheat expression database and visualized TPM values with a heat map (Figure S2). The results showed that *TaELP3-A, TaELP3-B, TaELP3-D*, and *TaELP5-D* were expressed at high levels in roots, stems, leaves, and inflorescences at various stages. In addition, we found that *TaELP5-D* was upregulated in flag leaves with the prolongation of postflowering time, while *TaELP3-A*, *TaELP3-B*, and *TaELP3-D* were gradually downregulated in flag leaves. Therefore, we speculated that *TaELP* genes may be related to senescence. However, *TaELP1-D* and *TaELP6-A* had lower expression levels in all different tissues and developmental stages of wheat. *TaELP4-A*, *TaELP4-B,* and *TaELP4-D* were found to be at higher levels expressed in the root, stem, leaf, and spike tissues. *TaELP1-A*, *TaELP1-B*, *TaELP2-A*, *TaELP2-B*, *TaELP2-D*, *TaELP5-A*, *TaELP5-B*, *TaELP6-B*, and *TaELP6-D* had tissue expression specificity in the stem, milk grain stage, stem, spike, leaf at seven-leaf stage, and roots at the three-leaf stage.

*TaELP3-A*, *TaELP3-B*, *TaELP3-D*, and *TaELP5-D* had the upregulated expression at 6 h after drought treatment (Figure S3). *TaELP3-A*, *TaELP3-B*, and *TaELP3-D* had higher expression in low-temperature stress. Under salt stress, the expression of *TaELP3-A*, *TaELP3-B*, and *TaELP3-D* were all upregulated, whereas *TaELP4-A*, *TaELP4-B*, and *TaELP4-D* had the highest upregulated expression after salt stress at 48 h. *TaELP5-D* showed very low expression under drought and low-temperature stress but showed an upregulated expression pattern at the early stage of salt stress. The expression patterns of *TaELP2-B* genes showed an upregulated trend, and the upregulated expression was most obvious at 48 h under salt stress (Figure S3). Furthermore, other *TaELPs* showed either no expression or downregulated expression patterns under different stress conditions in wheat.

### 2.9. Expression Pattern Validation Analysis of TaELPs

**Figure 7:**
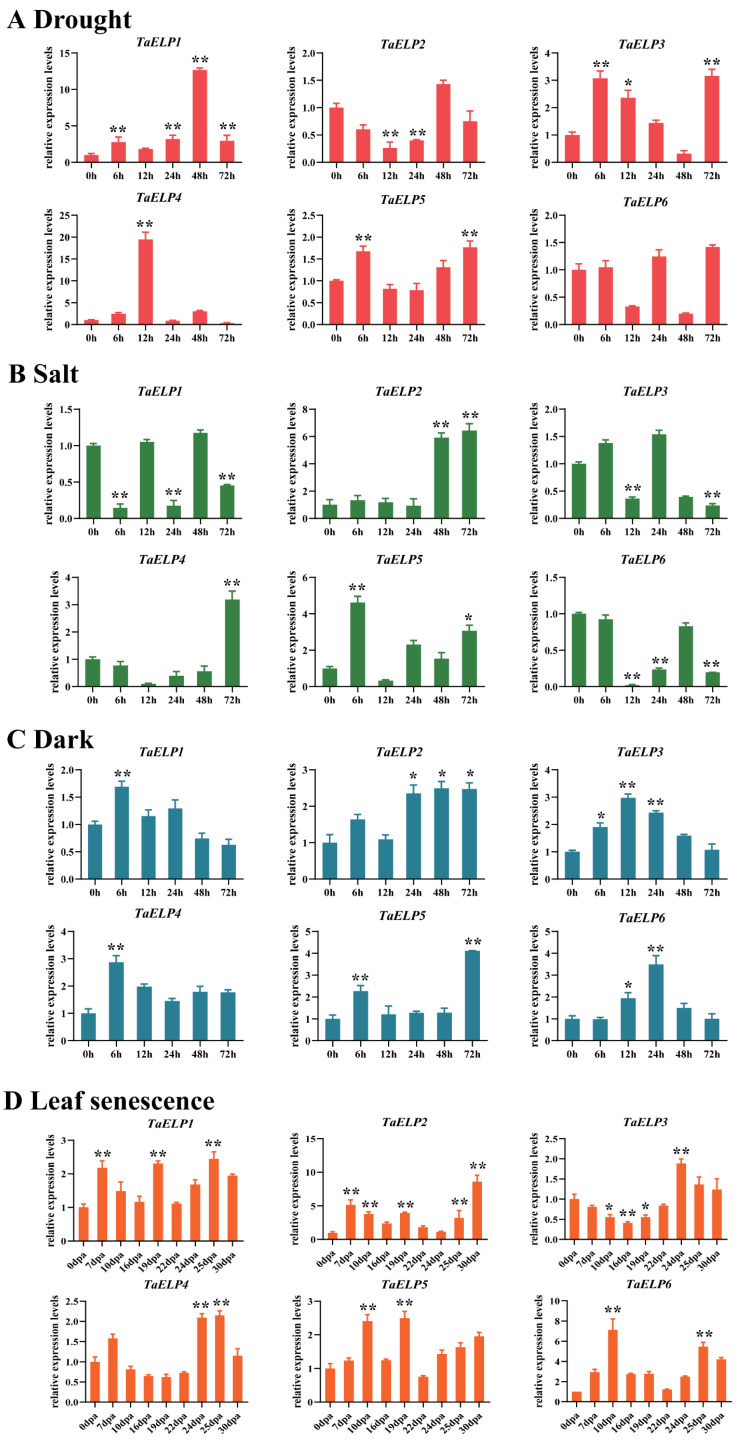
Relative expression profiles of *TaELPs* in response to (**A**) drought stress, (**B**) salt stress, (**C**) dark stress, and (**D**) leaf senescence. The 0 h post-treatment for A, B, and C or 0 days after anthesis for D were used as a control. The relative transcripts of all genes were analyzed using qRT-PCR. The relative transcript levels of *TaELPs* were measured using the comparative threshold (2^−ΔΔCT^) method. Data normalized with the transcripts of wheat elongation factor *TaEF-1α*. The 0 h post-treatment and 0 days after anthesis was used as a control and standardized with 1. Values represent the mean ± SE from three independent biological samples. Asterisks (*p* < 0.05) or double asterisks (*p* < 0.01) designate significant differences from 0 hpt or 0 days after anthesis by the Student’s *t*-test.

To further understand the potential response mechanism of the wheat *TaELP* gene family in tolerance to abiotic stress, hormone response, and leaf senescence, we analyzed the expression patterns of all *TaELP* genes under abiotic stresses (drought, salt, and dark treatment), during hormone treatments (IAA, SA, ABA), and during leaf senescence. Under drought treatment, *TaELP2* was significantly downregulated at 12 h and 24 h after drought treatment, and *TaELP6* was downregulated at 12 h and 48 h after drought treatment. The expressions of other *TaELP* genes were upregulated to various degrees at different time points after drought treatment. Among them, *TaELP1*, *TaELP3*, and *TaELP4* were significantly upregulated, and *TaELP3’s* significant upregulation was observed after 6 h and 72 h of drought treatment. The overall trend of *TaELP1* was upregulated, and significant upregulation was observed at 6 h and from 24 h to 72 h after drought treatment. The expression of *TaELP4* was most significantly upregulated at 12 h. *TaELP5* exhibited an overall upregulated expression trend, and the expression was significantly upregulated at 6 and 72 h after drought stress (Figure 7A).

Under salt stress, compared with the control, *TaELP1* was significantly downregulated at 6 h, 24 h, and 72 h. The expression level of *TaELP2* was upregulated, and the up-regulation was most obvious at 48 h to 72 h. *TaELP3* was significantly downregulated at 12 h and 72 h after salt treatment. *TaELP4* expression was downregulated at the early stage after salt stress but was significantly upregulated at 72 h. Compared with the control, *TaELP5* expression was upregulated at most of the time points and significantly upregulated at 6 h and 72 h of salt treatment. *TaELP6* was significantly downregulated at 12 h, 24 h, and 72 h after salt treatment (Figure 7B).

To explore the induced expression pattern of all members of the *TaELP* family in plant leaf senescence, we performed dark treatments at different times (Figure 7C). The results showed that the expressions of all *TaELP* genes were upregulated to various degrees at the early or late stage of dark treatment. For example, the expression pattern of the *TaELP2* gene showed an upregulated trend, and *TaELP2* was significantly upregulated at 24 h, 48 h, and 72 h of dark treatment. *TaELP1* and *TaELP4* were significantly upregulated at 6 h of dark treatment. The expression of *TaELP3* was significantly upregulated from 6 h to 24 h of dark treatment. *TaELP5’s* significant upregulation was observed at 6 h and 72 h after dark treatment. TaELP6 was significantly upregulated at 12 h and 24 h after dark treatment (Figure 7C). Moreover, during natural leaf senescence, *TaELP1* was significantly upregulated at 7, 19, and 25 days after anthesis; *TaELP2* was most significantly upregulated at 30 days after anthesis; *TaELP3* was significantly downregulated at 10, 16, and 19 days after anthesis; *TaELP4* was significantly upregulated at 24 and 25 days after anthesis; and *TaELP5* and *TaELP6* were significantly upregulated at 10 days after anthesis (Figure 7D), which suggests a significant role of *TaELPs* during the wheat leaf senescence process.

Under IAA treatment, the expression patterns of most *TaELP* genes were downregulated, and only *TaELP3* and *TaELP5* genes were upregulated. *TaELP3* was most significantly upregulated after 48 h of IAA treatment; *TaELP5* was upregulated immediately after 6 h of *IAA* treatment, and the expression level reached a peak and then showed a downward trend (Figure S4A). Under SA treatment, it was observed that only *TaELP5* exhibited low expression levels at all time treatment stages, and other *TaELP* genes were upregulated to varying degrees. For example, *TaELP2* was significantly upregulated in the early and late stages of SA treatment. The gene expression patterns of *TaELP4* and *TaELP6* showed an overall upward trend and were significantly upregulated after 12 h of SA treatment. The transcription levels of *TaELP3* and *TaELP1* increased first and then decreased and were significantly upregulated after 24 h of SA treatment (Figure S4B).

Under ABA treatment, the relative expression levels of all *TaELP* genes were significantly different (Figure S4C). *TaELP6* showed a significant upregulation at all time treatment stages compared to the control. *TaELP1*, *TaELP2*, *TaELP4*, and *TaELP5* showed an overall upward trend, and the gene expression was upregulated most significantly after 72 h of treatment. Moreover, *TaELP3* showed a significant upregulation only at ABA treatment at 6 h compared to the control (Figure S4C). Overall, our expression analysis results suggested that the members of the wheat *TaELP* gene family might play important regulatory roles in abiotic stresses, leaf senescence, and hormone signaling pathways.

### 2.10. Phenotypes of TaELP2-Silenced Wheat Seedlings in Dark-Induced Leaf Senescence

**Figure 8:**
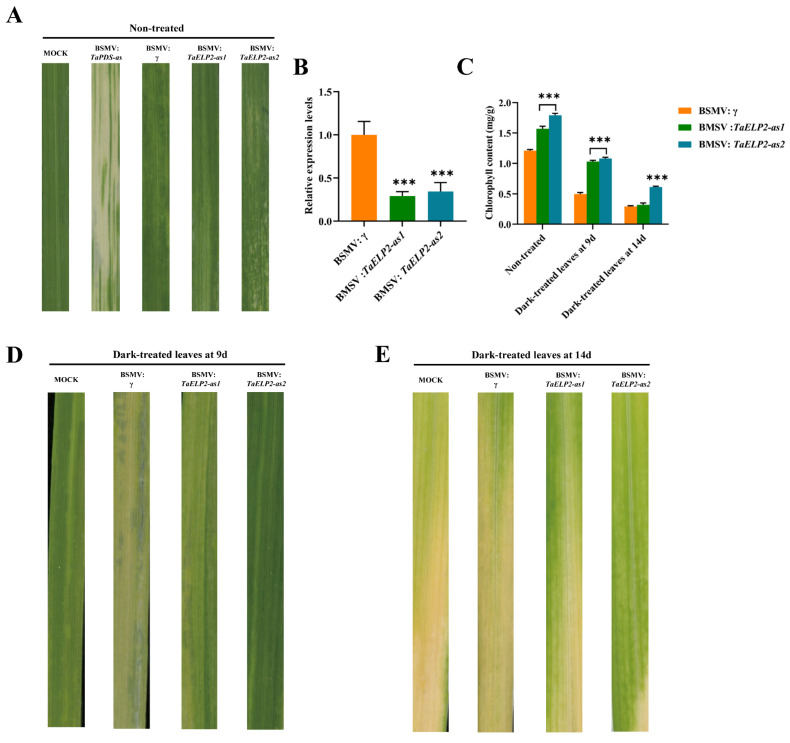
BSMV-mediated gene silencing of *TaELP2* delays dark-induced leaf senescence in wheat. (**A**) Virus symptoms after ten days of BSMV virus inoculation. (**B**) Silencing efficiency analysis of *TaELP2* in BSMV-inoculated leaves. The relative expression of *TaELP2* was analyzed by using qRT-PCR and calculated using the comparative threshold (2^−ΔΔCT^) method. Data normalized with the expression of wheat elongation factor, *TaEF-1α*. The BSMV-γ was used as a control and standardized with 1. Values represent the mean ± SE from three independent biological samples. Triple asterisks (*p* < 0.001) designate significant differences from BSMV-γ using the Student’s *t*-test. (**C**) Changes in chlorophyll content of BSMV-infected wheat leaves after dark treatment at 0, 9, and 14 days. Values represent the mean ± SE from three independent biological samples. Triple asterisks (*p* < 0.001) designate significant differences from BSMV-γ using the Student’s *t*-test. (**D**,**E**) Phenotypes of wheat leaves after dark treatment at 9 and 14 days, respectively.

By analyzing the expression pattern of *TaELPs* under dark and natural senescence, we found that the *TaELP2* gene may be involved in the regulation of wheat leaf senescence. To clarify the function of the wheat *TaELP2* gene in leaf senescence, the *TaELP2*-specific fragments were inserted into the original BSMV:γ genome, and the gene was silenced by virus-induced gene silencing technology, followed by dark treatment to observe its phenotypic changes. The BSMV:γ was used as a negative control, and BSMV:*TaPDS-as* was used as a positive control, in which leaf bleaching could be observed after silencing (Figure 8A). At ten days postinoculation, mosaic symptoms of the virus were observed in virus-inoculated plants, but further leaf growth was not affected. In contrast, BSMV:*TaPDS-as*-inoculated plants gradually displayed photobleaching symptoms (Figure 8A), suggesting that the virus-induced gene silencing was successfully achieved. To verify whether the *TaELP2* gene was effectively silenced, the silencing efficiency of *TaELP2* was analyzed with qRT-PCR (Figure 8B). The results showed that the expression level of *TaELP2* in the plants infected with BSMV:*TaELP2-as1* and BSMV:*TaELP2-as2* was significantly decreased compared with the BSMV:γ-infected plants. The chlorophyll content of the *TaELP2*-silenced and control leaves at 0 d, 9 d, and 14 d after dark treatment were measured, and the chlorophyll content of the *TaELP2*-silenced leaves was significantly higher than that of the control (Figure 8C). Then the wheat leaf phenotypes of the negative control and silenced plants were observed after dark treatment at 9 d and 14 d, and it was found that the leaves of *TaELP2*-silenced plants showed a stay-green phenotype compared to those of the negative control (Figure 8A). Therefore, the above results indicated that *TaELP2* gene silencing may delay leaf senescence, which may further clarify how *TaELP2* affects the leaf senescence process in wheat.

### 2.11. Prediction of Protein–Protein Interactions of Wheat ELPs

To study the interaction between wheat *TaELPs* and other proteins, a network was constructed using the STRING database (Figure S5 and Table S9). Based on the predicted results, we observed that TaELP1, TaELP2, TaELP3, and TaELP6 had protein interactions with a Chromatin-associated protein KTI12 (Traes_5BL_92F800E16.1, Traes_5BL_D8ECD483D.2 and Traes_5DL_A9A62BF38.1) and a Diphthamide biosynthesis protein 3 (Traes_7BL_69CD9E49D.2, Traes_7DL_EF5C1F9EA.1). In addition, TaELP3 had protein interactions with Traes_2AS_03ED0D137.1, Traes_2BS_E0BE8F2D1.1, and Traes_2DS_E75C5D4AC.1, which encodes WD40 repeat-containing proteins. No protein interacting with TaELP4 and TaELP5 was found.

WD repeats proteins are widely present in eukaryotes and are involved in various cellular behavioral and physiological regulations, such as signal transduction, activation of transcriptional activity, cell growth and development, and control of apoptosis. The presence of WD40 domains or repeated WD40 motifs can act as a scaffold for protein–protein or protein–DNA assembly, play an important role in protein interactions, and act as a mediator of transient interactions between other proteins [44]. Furthermore, chromatin-associated protein KTI12 was found to interact with the Elongator complex (ELP) in the process of RNA polymerase II-promoting transcription elongation [45].

## 3. Discussion

Global warming is a common challenge for global agricultural development. According to the prediction of climate change models, global crop yields have been declining as the climate changes, but the decrease in wheat yield is closely related to abiotic stresses [46]. One of the effective ways to deal with the current bottleneck of wheat production is to tap more wheat stress resistance gene resources and apply for the breeding of wheat-resistant varieties. Elongator complex (*ELP*) is an indispensable component of gene transcription in eukaryotes, and it can also indirectly participate in cell behaviors such as extracellular secretion, telomere gene silencing, and DNA damage repair by modifying tRNA to participate in the translation process, which influences abiotic and biotic stress responses as well as plant growth and development [6].

### 3.1. Evolution and Genetic Relationship of TaELPs

In higher plants, there was a gap between the number of *ELP* gene family members in closely genetic relationship plants. A total of six *ELP* genes were identified in *Arabidopsis*, and six members were identified in rice [18,47]. Compared with *Arabidopsis*, rice is more closely related to wheat, but the number of rice *ELP* gene family members is lower than the number of wheat *ELP* genes. In the process of studying plant evolution, it is found that the expansion of gene families is closely related to the occurrence of gene duplication, which may originate from segmental, tandem, or whole-genome duplication [35,41,48,49]. Segment duplication (SD) is common in biological evolution. When the repetitive DNA sequence exceeds 1kbp or the identity is higher than 90%, we consider that the gene has SD on the chromosome [50]. In this study, it was found that the members of the wheat *TaELP* gene family contained 18 pairs of *ELP* paralogous genes, all of which originated from large segment duplication. Two fragment duplications were observed on 2A, 2B, 2D, 4A, 4B, and 4D, and one fragment duplication was observed on 1A, 1B, 1D, 7A, 7B, and 7D (Figure 1 and Table S2). These results suggest that fragment duplication may have a dominant role in the evolution and expansion of the wheat *TaELP* gene family. The evolutionary selection pressure (Ka/Ks) and divergence time (MYA) of 18 pairs of *TaELP* genes were calculated, and the Ka/Ks < 1 of all duplicated gene pairs indicated that *TaELPs* belonged to purifying selection in long-term evolution. The phylogeny and cluster analysis of wheat *TaELP* proteins showed that wheat *ELP* proteins were more closely related to the evolution of monocotyledonous plants.

In addition, to further understand the genetic relationship of the wheat *TaELP* gene family, we investigated the collinearity relationship of *ELP* genes in wheat and other species (including monocotyledonous and dicotyledonous plants). None of the wheat orthologous gene pairs were found in *Arabidopsis*, only three *ELP* orthologous gene pairs were found in *Glycine max*, and most wheat orthologous gene pairs were found in *Oryza sativa*, *Brachypodium distachyon*, *Triticum dicoccoides,* and *Aegilops tauschii*. It has been shown that allohexaploid bread wheat (AABBDD) was produced by crossing allotetraploid *Triticum dicoccoides* (BBAA) with the diploid *Aegilops tauschii* (DD) containing the D genome [51]. Therefore, we speculated that hybridization was part of the reason for more orthologous gene pairs between wheat and *Triticum dicoccoides* and *Aegilops tauschii*. The 56 orthologous gene pairs identified in wheat were all derived from whole-genome duplication or fragment duplication within the genome. We also calculated evolutionary selection pressure (Ka/Ks) and divergence time (MYA) between wheat and other species, with Ka/Ks < 1 for all duplicate gene pairs, suggesting that *TaELPs* belonged to purification selection in long-term evolution. Therefore, results suggested that *TaELP* genes in wheat may have evolved from orthologous genes in other plant species and are more closely related to monocotyledonous plants.

### 3.2. Structural Diversity of Wheat TaELPs 

The protein conserved domain analysis showed (Figure 5) that each *ELP* subunit protein in wheat contains its corresponding conserved domain, which further illustrated the evolutionary and functional conservation of eukaryotic *ELPs* [6]. Compared with the previously reported domains of *Arabidopsis*, rice and wheat *ELPs*, we found that there were significant differences in the number of domains between some subunits, such as OsELP2 and AtELP2, which contained five and six WD40 repeat protein domains, respectively, and OsELP6 and AtELP6 contained two and one Elongation complex protein 6 domains, respectively, [9,18], while wheat contained one Elongation complex protein 6 domain and four WD40 repeat protein domains, although the size of the domains was similar. It showed that in different plant species, there were slight differences between the structure and number of *ELPs*, suggesting that there may be a change in biological function. The WD40 repeat protein domain contained in ELP2 has been widely reported to participate in a variety of biological processes of plant growth and development and play an important role in protein–protein and protein–DNA interactions. Plant anthocyanin biosynthesis and abiotic stress response are closely related [44]. IKI3 is a chromatin-associated domain that interacts with *ELP* and contains the WD40 repeat protein during RNA polymerase II-promoted transcription elongation [3]. ELP6 is an accessory subunit of the Elongator complex, is associated with histone acetylation in the nucleus and tRNA modification in the cytoplasm, and is able to catalyze the elongation of transcription by RNAII [52]. PAXNEB has been found in different eukaryotes. It is a component of the RNA polymerase II extension protein subunit and HAP subcomplex, which can catalyze intracellular histone acetylation [5,7,14,53]. Members of the wheat *TaELP* gene family have different domains, suggesting that the six subunits have different potential functions in plants.

The gene structure and conserved motif analysis of the wheat *TaELP* genes showed (Figure 4) that there were quantitative differences in the exon–intron number of the *TaELP* genes, which was similar to the results in *Arabidopsis* and rice [18]. Among the 10 identified motifs, all members of the wheat *TaELP* gene family contained a conserved motif of MOTIF2. These results suggested that the evolutionary pattern of the wheat *TaELP* gene family was relatively conserved. Further, subcellular localization prediction showed that most members of the wheat *TaELP* gene family were in the cytoplasm, and a few were in the nucleus, plastid, and plasma membrane. Subcellular localization results of the Elongator complex have been reported in a variety of plants. Nelissen and colleagues [54] detected GFP-ELO3 fusion protein in the nucleus using the GFP fusion protein method. Two years later, Tran et al. found ELP3 red fluorescent protein under the detection of confocal laser scanning microscopy through the epidermal cells of fava bean leaves, and the results again showed that the Elongator complex exists in the nucleus and cytoplasm [55]. In conclusion, most of the above reports have confirmed that the elongator complex (*ELP*) is localized in the nucleus and cytoplasm, which was also confirmed by our prediction of the subcellular localization of all *TaELP* genes in wheat.

### 3.3. Transcription Analysis of TaELP Genes Reveals Its Role in Wheat Growth, Development, and Abiotic Stress Tolerance

Different CAREs’ distributions in promoter regions may indicate variations in gene regulation and function [35,56]. Through the analysis of 2000 bp cis-acting elements upstream of the promoter of wheat *TaELP* gene family members, we can further understand the process of *TaELP* gene-regulating physiological changes in wheat. We found that all *TaELP* genes contain more than one cis-acting element in response to abiotic stress. The frequency of drought-induced cis-elements MYC, MYB, MBS, etc.; stress defense response cis-elements STRE, as-1, as-1, ARE, etc.; and other stress-related CAREs in all *TaELP* genes (Figure 6 and Table S7) is very high [56]. MYB and MYC transcription factor binding elements were involved in plant responses to drought, high salt, and low temperature, and they regulate the expression of related genes under stress [57,58]. In the study of drought resistance of various crops, it was found that MBS elements generally exist in the promoter sequences of drought resistance-related genes and are closely related to the response to drought [59] indicating that they play an important role in abiotic stress. In addition to the above abiotic stress response elements, they also contained defense and stress response elements and a variety of biological stress-related transcription factor binding elements. We also found hormone-related elements, including ABRE (abscisic acid-responsive), TCA-element (salicylic acid-responsive), CGTCA-motif (MeJA responsive), p-box, TATC-box (gibberellin responsive element), etc. ABRE response element was related to ABA-related gene expression, which was regulated by ABA-dependent or ABA-independent factors in abiotic stress [60], indicating that *TaELPs* may be involved in signaling during the wheat stress response. In addition, the frequency of many cells cycle-related CAREs and CAREs with unknown functions was also high, indicating that *ELP* genes in wheat have different functions during wheat development. The cis-acting elements of the *ELP* family genes were closely related to their possible physiological processes; predicting the cis-acting elements of unknown gene families is helpful to quickly predict gene functions. It is an effective way to screen candidate genes and analyze gene functions using reverse genetics [18,61].

The tissue expression patterns of genes are often closely related to their gene functions. The 18 *TaELP* genes were expressed to various degrees in each stage of seedling, root, stem, leaf, and panicle, indicating that they may play an important role in the growth and development of wheat plants [62]. *TaELP2*, *TaELP3*, *TaELP4*, and *TaELP5* were highly expressed in roots, stems, leaves, and ears at different developmental stages, and they had different degrees of response under different abiotic stress treatments, such as drought, heat shock, high salt, and low-temperature environments, indicating that *TaELP2*, *TaELP3*, *TaELP4*, and *TaELP5* may initiate defense systems in different organs and tissues to adapt environmental stress. In the senescence stage of wheat, we also found that *TaELP2*, *TaELP3*, *TaELP4*, and *TaELP5* were expressed in flag leaves after different flowering stages, indicating that these genes may be related to the senescence process of wheat.

The results of the cis-acting elements and expression pattern prediction analysis suggested that *TaELP* family genes may play an important role in wheat resistance to abiotic stress and growth and development. To verify the above prediction analysis, we further analyzed the induced expression patterns of *TaELP* genes under three abiotic stresses (drought, salt, and dark), three hormone treatments (IAA, SA, and ABA), and different senescence stages of wheat. The qRT-PCR results showed that the expression levels of *TaELP1*, *TaELP3*, and *TaELP4* were significantly increased under drought stress, which may be related to the fact that the promoter region contained multiple cis-acting elements related to drought response, such as ABRE, MYB, and MYC. Under salt stress treatment, the results of qRT-PCR showed that the expression levels of *TaELP2* and *TaELP4* were significantly increased during 48–72 h of salt stress, and the expression levels of *TaELP5* were significantly upregulated in the early and late stages of salt stress (Figure 7B). Studies have shown that Elongators play an important role in plant growth and development. In the phenotypic observation of *Arabidopsis elo*/*Atelp* mutants, it was found that the germination and seed setting rates at the seedling stage were lower, the germination was slower, the flowering was delayed, the morphological structure of leaves and flowers was abnormally developed, and the leaf area was reduced. The apical meristem develops irregularly, and the underground taproot and hypocotyl grow slowly [20,22,54]. This study found that all *TaELP* genes were highly expressed in flag leaves at different time points after flowering (Figure 7D), and their transcription levels were significantly increased under dark stress (Figure 7C). *TaELP2* was significantly upregulated under dark stress and at the late stage of senescence, indicating that *TaELP* genes were closely related to wheat senescence and that *TaELP2* might play an important role in wheat senescence (Figure 7D). Further, the *TaELP2* gene was silenced in wheat using BSMV-VIGS technology, and the phenotype of silenced wheat plants was observed after dark treatment (Figure 8). The silenced plants showed a stay-green phenotype, and the chlorophyll content was higher than that of the control. Therefore, *TaELP2* might be involved in regulating the senescence of wheat. However, its specific biochemical function is still unclear and needs further research.

Cell proliferation is closely related to Elongator, and cell proliferation is often induced by plant hormones [15,63]. When studying the expression of *ELP*-related genes in different plants, it was found that a large number of auxin genes have high differential expression, so we speculate that Elongator may induce the expression of auxin-related genes to control plant growth and development [54]. Therefore, we performed the expression analysis of all *TaELPs* under three hormone treatments (IAA, SA, and ABA). Further studies found that auxin-related genes were hypoacetylated at histone H3K14, suggesting that the Elongator complex may interact with RNAPII to catalyze the formation and transcription of chromatin, thereby promoting the expression of auxin-related genes [23,54]. Therefore, we treated all *TaELPs* with IAA at different times and found that only *TaELP3* had a higher expression, suggesting that *TaELP3* may be an important regulator of wheat growth and development (Figure S4). Salicylic acid (SA) was a signaling molecule that initiates stress response mechanisms when plants encounter pathogens [64]. SA accumulates after pathogen infection and is critical for activating local and systemic acquired resistance [65]. Elongator inhibits the expression of CAT3 and other related antioxidant genes and can promote the expression of SA-related genes [15,19]. In all *TaELPs* treated with SA at different times, all except *TaELP5* were expressed, and *TaELP2* was significantly expressed, indicating that wheat *TaELP2* may play an irreplaceable role in pathogen defense (Figure S4). Under ABA stress treatment, the expression of *TaELP3* was not significantly upregulated, and the remaining *TaELP* genes were upregulated to varying degrees under ABA treatment at different times (Figure S4). We found that *TaELP2*, *TaELP4*, *TaELP5,* and *TaELP6* were significantly upregulated in the early and late stages of ABA stress treatment. Plant hormone ABA can cause plant cell behavior regulation and physiological signal transduction and affect different stages of plant growth and development. When subjected to abiotic stresses such as drought, salt, and high temperature, ABA can conduct signals to activate plant stress response mechanisms [19,66,67,68,69]. According to related reports, ABA signaling molecules were involved in RNA-related processes such as RNA splicing, RNA structure stabilization, and RNA elongation, indicating that ABA signaling was closely related to RNA metabolism and regulation [19,24,70,71,72,73,74]. ABA signaling was associated with the histone acetyltransferase Elongator complex [24]. Therefore, we analyzed the relationship between ABA stress and the three abiotic stresses. The results showed that the expression levels of *TaELP2* and *TaELP4* were significantly increased at 48–72 h induced by salt stress and ABA, and the expression levels of *TaELP5* were significantly increased at 6 and 72 h. indicating that *TaELP2*, *TaELP4*, and *TaELP5* genes play an important role in the regulatory mechanism of plants resisting salt stress and may exist in the salt stress response signaling pathway dependent on ABA regulation. All the above results showed that *TaELP2*, *TaELP3*, *TaELP4*, and *TaELP6* may be important regulators of abiotic stress and leaf senescence in wheat, and they play an important role in signal transduction. Therefore, this temporal and spatial expression pattern of *TaELP* genes indicates that these *ELPs* might have a function in different tissues and various developmental stages as well as abiotic stress tolerance in wheat.

Through predictive analysis of interactions between wheat *TaELPs* and other proteins (Figure S5, Table S9), we found that *TaELPs’* interacting proteins play roles in plant growth and development and hormone and abiotic stress responses. The three interacting proteins contained the WD40 domain. The WD-repeat protein can be used as a scaffold for protein–protein assembly and may interact with *TaELPs* to play a role in plants, which can regulate plant growth and development, transcriptional regulation, and hormone signaling, and initiate plant stress defense mechanisms [44,75]. The WD40 repeat protein had been extensively studied in *Arabidopsis*, and it can act as a regulator of plant growth and development to regulate plant-specific development [75]. Chromatin-associated protein KTI12 is an important regulator of the Elongator complex and is involved in the modification of uridine bases in eukaryotic tRNA, and there is a close physical and functional relationship between them [45,76]. Two stress-related genes *PtKTI12A* and *PtKTI12B* were identified in *Populus trichocarpa* under high temperature and drought stress, and their expression levels were analyzed in each specific tissue, and it was found that they were differentially expressed [77]. The results showed that in the stress response mechanism, KTI12 protein can be induced to express and activate plant resistance to stress and participate in tRNA swing uridine modification [77]. Thus, *TaELPs* along with their interacting partners might be required to develop wheat stress tolerance. Overall, these findings are useful in elucidating the specific biological activities of *TaELP* genes to generate high-yielding and stress-tolerant wheat cultivars.

## 4. Materials and Methods

### 4.1. Identification of Wheat ELPs Family Gene Members

We used 12 *ELP* genes from *Arabidopsis* and rice to find members of the *ELP* gene family in the wheat genome [18,47]. The *ELP* protein sequences of *Arabidopsis* and rice were retrieved from the *Ensembl Plants* database. *ELP* genes were identified from the whole wheat genome using BLASTp against the most recent wheat entire genes from IWGSC (RefSeq v1.0) (http://plants.ensembl.org/index.html, E-value < 10^−5^ and bit-score > 100, accessed on 4 June 2021) and through the Blast Compare Two Seqs tool of TBtools (v1.09832) [78]. Finally, after eliminating duplicated sequences, the output of BLASTp and TBtools were selected for domain analysis. SMART (http://smart.embl-heidelberg.de/, accessed on 14 June 2021) or InterPro (https://www.ebi.ac.uk/interpro, accessed on 14 June 2021) or NCBI CDD (https://ncbi.nlm.gov.Structure/cdd/cdd.shtml, accessed on 14 June 2021) and HMM scan (https://www.ebi.ac.uk/Tools/hmmer/search/hmmscan, accessed on 14 June 2021) were utilized to check for the existence of *ELP* gene family domains in the remaining sequences. The length, molecular weight, isoelectric point (pI), and gross average value (GRAVY) of the wheat *ELP* proteins were calculated using ProtParam (v3.0) software (https://web.expasy.org/protparam/, accessed on 30 June 2021).

### 4.2. Sequence Alignment and Phylogenetic Tree Construction

Full-length protein sequences from several species were aligned using ClustalW in MEGA X [79], and all sequences after alignment analysis were imported into MEGA X for constructing a phylogenetic tree using the neighbor-joining (NJ) method [80] with 1000 bootstrap values [81].

### 4.3. Chromosomal Localization, Gene Duplication, and Collinearity Analysis

For chromosomal distribution, *ELPs’* genomic positions were obtained from the Ensembl Plants BioMart (http://plants.ensembl.org/biomart/martview/, accessed on 6 July 2021). The *ELPs* were numbered according to their ascending chromosomal location and were given a “Ta” prefix. TBtools was used to visualize the *TaELPs* on the wheat chromosomes. McscanX software was used to investigate tandem and segmental duplications within the *TaELP* gene family and collinearity between *ELPs* from wheat and several other species [82]. The TBtools were used to compute the nonsynonymous substitution rate (Ka), synonymous substitution rate (Ks), and the Ka/Ks ratio, and the divergence time T was estimated with T = Ks/(2 × 9.1 × 10^−5^)Mya [78].

### 4.4. Subcellular Localization and 3D Structure Modeling

Subcellular localization was predicted using WoLF PSORT (https://wolfpsort.hgc.jp/, accessed on 20 July 2021) and CELLO v.2.5 (http://cello.life.nctu.edu.tw, accessed on 20 July 2021). The SWISS-MODEL (https://swissmodel.expasy.org/interactive#sequence, accessed on 30 July 2021) was used to create a three-dimensional (3D) protein structure of *TaELPs*. Protein secondary structure analysis used SOPMA software (https://npsa-prabi.ibcp.fr/cgi-bin/secpred_sopma.pl, accessed on 3 August 2021).

### 4.5. Structure, Domain, and Motif Analysis of TaELP Genes

Wheat genome annotation file (GFF3 format), CDS sequence, and protein sequence of *ELP* genes were retrieved from the EnsemblPlants database (http://plants.ensembl.org/index.html, accessed on 20 August 2021). The gene-related information of *TaELPs* was extracted using the function of Gtf/Gff3 Sequences Extract of TBtools [78]. The Gene Structure Display Server 2.0 (http://gsds.gao-lab.org/, accessed on 27 August 2021) was used to visualize intron, exon, and untranslated regions. The *ELP* gene domains were retrieved from the Pfam database, and Evolview (https://www.evolgenius.info/evolview-v2/, accessed on 2 September 2021) was used to visualize the gene domains. To anticipate the conserved motifs of *TaELPs*, we employed the motif-based sequence analysis tools MEME version 5.4.1 (http://meme-suite.org/tools/meme, accessed on 10 September 2021) with a maximum section of up to 10 motifs and visualized using TBtools [78].

### 4.6. Analysis of Cis-Acting Regulatory Elements (CAREs) and Protein Interaction Networks

The 2000 bp upstream sequences of 18 *TaELP* genes were retrieved from the Ensembl Plants database (http://plants.ensembl.org/index.html, accessed on 20 September 2021) and submitted to the online software PlantCARE (http://bioinformatics.psb.ugent.be/webtools/plantcare/html/, accessed on 21 September 2021) to find CAREs. Subsequently, the TBtools Heatmap was used for data visualization [78]. The STRING online server (https://string-db.org/cgi, accessed on 30 September 2021) was used to predict the protein interaction network of the *TaELPs*.

### 4.7. Tissue-Specific Expression and Stress Analysis of Wheat TaELPs

The wheat (Chinese spring) RNA-Seq data was retrieved from the Wheat Exp database (http://wheat-expression.com/, accessed on 1 October 2021), and the TPM (transcripts per million reads) value was used to evaluate the transcript abundance of wheat *TaELP* genes. The MeV tool was used to visualize the expression [83].

### 4.8. Plant Materials and Treatments

Wheat cultivar, Jinmai 39, was selected to study the expression of *TaELPs* under abiotic stress (drought stress, salt stress, dark stress) and hormone treatments (ABA, IAA, SA). The seedlings were grown in an artificial climate box (16 h light/8 h dark) at 22 °C. The wheat plants were grown to the 2–3 leaf stage, and 2–3 leaf stage seedlings were treated with sterile ddH_2_O (control) or 20 % PEG-6000 solution for drought treatment, and samples were obtained at 0, 6, 12, 24, 48, and 72 h after treatment. Salt stress treatment was performed with sterile ddH_2_O (control) or 250 mM NaCl solution on 2–3 leaf stage seedlings, and samples were obtained 0, 6, 12, 24, 48, and 72 h after treatment. For dark stress, the 2–3 leaf stage seedlings were placed in a growth chamber with 0 Lux light intensity, and leaf samples were collected at 0, 6, 12, 24, 48, and 72 h after treatment. For abscisic acid (ABA), auxin (IAA), and salicylic acid (SA) treatments, wheat plants at the same stage were sprayed with 100 mM ABA or 100 mM IAA or 100 mM SA or 0.1 % (*v*/*v*) ethanol (control), and samples were obtained at 0, 6, 12, 24, 48, and 72 h after treatment. Yannong 19, a delayed senescence wheat cultivar, was cultivated in the field, and flag leaf samples were taken at 0, 7, 16, 19, 22, 24, 25, and 30 days after anthesis. All leaves collected were immediately frozen in liquid nitrogen and stored in a −80 °C freezer for further RNA extraction. Each of the above experiments was set up with 3 sets of repetitions.

### 4.9. Virus-Induced Gene Silencing (VIGS) and Chlorophyll Content Determination

The cDNA sequences of *TaELP2* were analyzed using SiFi12 software, and two segments with high and specific siRNA numbers were selected for gene silencing, *TaELP2-as1* and *TaELP2-as2*, respectively. Specific primers (Table S10) were designed, and two specific cDNA fragments of *TaELP2* were obtained using PCR and constructed into BSMV:γ genome. Then, the seedlings (Yannong 19) at the two-leaf stage were inoculated using friction. After 10 days of inoculation, the virus phenotype of the leaves was observed. The plants with virus phenotype were identified with RT-qPCR and treated in dark. The leaf phenotype and chlorophyll content were observed at 9 days and 14 days after treatment. The determination method of chlorophyll content was from Porra et al. [84].

### 4.10. RNA Extraction, cDNA First-Strand Synthesis, and Real-Time PCR Analysis

The Quick RNA isolation Kit (Tiangen Biochemical Technology Co., Ltd., Beijing, China) was used to extract RNA according to the manufacturer’s instructions, and DNase I treatment was used to remove DNA contamination. Synthesis of the first strand of cDNA was carried out according to the instructions of the kit (Takara, Japan). To measure the expression of *TaELPs*, gene-specific primers were designed using WheatOmics 1.0 PrimersServer Database (http://202.194.139.32/PrimerServer/, accessed on 21 October 2021). qRT-PCR analysis was performed with specific primers (Table S10). qRT-PCR reactions were conducted using the following protocol: 95 °C for 2 min, followed by 40 cycles of 95 °C for 20 s and 60 °C for 20 s and 72 °C for 20 s. We verified the specificity of each primer’s amplicon via melting curve analysis, and the *Elongation factor 1a* (*TaEF-1a*) was used as an internal reference gene (GenBank accession no. Q03033) [85]. The threshold values (CT) were generated using the ABI PRISM 7500 system (Applied Biosystems, Foster City, CA, USA), and the transcription level of *TaELPs* was assessed using the comparative 2^−ΔΔCT^ method [86].

## 5. Conclusions

Wheat is a major grain crop and a staple meal all over the world. Therefore, researchers have intended to improve wheat production, quality, and different stress tolerance. The current study utilized biological information methods to identify *ELP* family genes in wheat and carried out a comprehensive and systematic analysis. The gene structure, amino acid motif, and subcellular localization prediction results of the *TaELP* gene family showed that the *ELP* gene in wheat is highly conserved. The homology analysis between wheat and other plant species showed that the *TaELP* genes had a close homology relationship with *Aegilops tauschii*. The cis-acting element analyzed predicts 14 different types of abiotic stress response elements and 13 hormone response-related CAREs. Transcriptome data combined with qRT-PCR results showed that the *TaELP* genes have a predominant role in wheat development and stress tolerance regulation. Transient silencing of *TaELP2* exhibited a stay-green phenotype, and the chlorophyll content was higher than that of the control. Therefore, the findings of this work will aid in understanding the role of *ELPs* in plant developmental processes and various stress situations, as well as their sequential implementation to increase yield and generate stress-tolerant wheat cultivars.

## Figures and Tables

**Table 1:** Detailed annotation of the *TaELPs* in wheat.

Gene Name	Gene ID	Splice	PC	ORF	Chromosome Location					Introns	Exons	Length	M.W.	PI	Instability	Aliphatic Index	GRAVY	SL Prediction
		Variant			Chr	Strand	Start	End	Chr Length			(aa)	(KDa)		Index			
TaELP1-A	TraesCS1A02G104700.1	1	II	2511	1A	reverse	100,291,939	100,297,147	594,102,056	10	11	836	91.40	6.26	44.07	84.67	−0.093	cytosol
TaELP1-B	TraesCS1B02G116100.1	1	II	2508	1B	reverse	136,647,720	136,652,235	689,851,870	9	10	835	91.36	6.23	42.10	86.05	−0.090	cytosol
TaELP1-D	TraesCS1D02G096900.1	2	II	2535	1D	reverse	83,724,571	83,729,227	495,453,186	9	10	844	92.51	6.25	43.44	83.98	−0.085	nucleus
TaELP2-A	TraesCS2A02G203700.1	1	III	3978	2A	reverse	179,670,464	179,675,987	780,798,557	5	6	1325	147.31	5.60	44.21	90.59	−0.163	cytosol, nucleus, plasma membrane
TaELP2-B	TraesCS2B02G231000.1	1	III	3975	2B	reverse	227,082,327	227,087,872	801,256,715	5	6	1324	147.14	5.52	44.05	90.95	−0.161	cytosol, nucleus, plasma membrane
TaELP2-D	TraesCS2D02G212000.1	1	III	3978	2D	forward	170,443,244	170,448,462	651,852,609	5	6	1325	147.17	5.57	42.54	90.60	−0.158	cytosol, nucleus, plasma membrane
TaELP3-A	TraesCS2A02G320900.1	1	I	1710	2A	forward	550,539,215	550,542,666	780,798,557	8	9	569	63.57	8.88	35.59	85.55	−0.310	cytosol
TaELP3-B	TraesCS2B02G361800.1	1	I	1710	2B	reverse	514,861,001	514,865,480	801,256,715	9	10	569	63.61	8.88	35.55	85.89	−0.307	cytosol
TaELP3-D	TraesCS2D02G341600.1	1	I	1710	2D	forward	436,369,113	436,372,717	651,852,609	8	9	569	63.58	8.88	35.55	85.55	−0.312	cytosol
TaELP4-A	TraesCS4A02G045700.1	1	V	1155	4A	forward	37,776,004	37,782,022	744,588,157	9	10	384	42.72	5.36	51.44	84.32	−0.397	cytosol
TaELP4-B	TraesCS4B02G259300.1	1	V	1155	4B	forward	526,726,516	526,729,679	673,617,499	9	10	384	42.71	5.38	53.49	83.05	−0.413	cytosol
TaELP4-D	TraesCS4D02G259200.1	1	V	1155	4D	reverse	428,719,059	428,727,934	509,857,067	9	10	384	42.71	5.30	52.06	85.34	−0.390	cytosol
TaELP5-A	TraesCS4A02G105200.1	1	VI	759	4A	forward	119,080,643	119,083,199	744,588,157	4	5	252	27.05	5.97	31.37	101.83	0.193	cytosol
TaELP5-B	TraesCS4B02G198800.1	1	VI	753	4B	reverse	427,496,766	427,499,136	673,617,499	5	6	250	27.08	5.98	33.83	99.52	0.159	cytosol
TaELP5-D	TraesCS4D02G199700.1	1	VI	765	4D	reverse	346,452,075	346,454,270	509,857,067	4	5	254	27.24	5.91	35.06	97.56	0.163	cytosol
TaELP6-A	TraesCS7A02G522900.2	2	IV	1152	7A	forward	705,684,956	705,687,644	736,706,236	7	8	383	41.20	8.69	53.26	80.84	−0.252	plastid
TaELP6-B	TraesCS7B02G439900.1	1	IV	1158	7B	forward	705,251,306	705,254,066	750,620,385	7	8	385	41.29	8.97	50.04	78.88	−0.250	plastid
TaELP6-D	TraesCS7D02G512100.1	2	IV	1155	7D	forward	613,867,768	613,870,602	638,686,055	7	8	384	41.18	8.69	47.94	79.35	−0.252	plastid

PC, Phylogenetic clade; ORF, Open reading frame; No, Number; bp, Base pair; Chr, Chromosome; aa, Amino acid; M.W., Molecular weight; Pi, Iso electric point; GRAVY, Grand average of hydropathy, SL, Subcellular localization.

## Data Availability

All needed genome sequences and genome annotation files of wheat were obtained from the *Ensemblplants* database (https://plants.ensembl.org/Triticum_aestivum/Info/Index, accessed on 4 June 2021). The transcriptome sequencing data of different tissues and various abiotic stresses used in this study were retrieved from the Wheat Exp database (http://wheat-expression.com/, accessed on 1 October 2021) and these RNA-sequencing reads of the Wheat Exp database were previously deposited with NCBI under accession codes PRJEB25639, PRJEB23056, PRJNA436817, SRP133837, PRJEB25640, and PRJEB25593. All data generated in this study are available in the public and also included in the article and its Additional files. In addition, all databases or online software are included in this published article and its Supplementary Materials.

## Supplementary Materials

The following supporting information can be downloaded at: https://www.mdpi.com/article/10.3390/plants12040952/s1, Table S1: ELP protein sequences identified from the wheat genome, Table S2: The synteny relationships of wheat *ELP* genes with different plant species, Table S3: Phylogenetic tree member with their gene ID, Table S4: Sequence identity and query cover of TaELP proteins, Table S5: Validation of TaELP protein structures, Table S6: Details of the calculated secondary structure elements TaELPs by SOPMA, Table S7: Cis-acting element regulation (CAREs) with sequences and functions, Table S8: Frequency of all identifiedCis-acting element regulation (CAREs) in different *TaELPs* promoters, Table S9: The protein-protein interaction network between TaELPs and other proteins in wheat, Table S10: List of primers used for *TaELPs* qRT-PCR analysis, Figure S1: Schematic illustration of 3-D protein structures and the Ramachandran plot for TaELP proteins. (A)TaELP proteins predicted three-dimensional structures. (B)The Ramachandran plot for TaELP proteins, Figure S2. Heatmap representing the expression patterns of TaELPs genes. (A)In different organs and developmental stages. (B) The expression of the *TaELP3* gene in different organs of wheat, predicted by Wheat eFP Browser. Figure S3: Heatmap representing the expression patterns of *TaELPs* genes. Under different abiotic stress conditions. The TPM values are used directly to build the heatmap. CS: Chinese Spring Na: salt stress h: hours, Figure S4. Relative transcript profiles of TaELPs in response to (A) Indole-3-acetic acid (IAA), (B) salicylic acids (SA), (C) abscisic acid (ABA) The relative transcripts of all genes were analyzed using qRT-PCR. Figure S5: Protein-protein interaction analysis of TaELPs proteins. Protein-protein interaction network generated by STRINGV9.1. Each node represents a protein and each edge represents an interaction, colored by evidence type.
