# Supplementary material for: Insights into the Bioinformatics and Transcriptional Analysis of the Elongator Complexes (ELPs) Gene Family of Wheat: TaELPs Contribute to Wheat Abiotic Stress Tolerance and Leaf Senescence
Source: Plants (Basel). 2023 Feb 20;12(4):952. doi: 10.3390/plants12040952 (PMC9961319; doi:10.3390/plants12040952)
Supplement: Supplementary file 1 [file plants-12-00952-s001.zip › Supplementary figures 02.17.pdf]

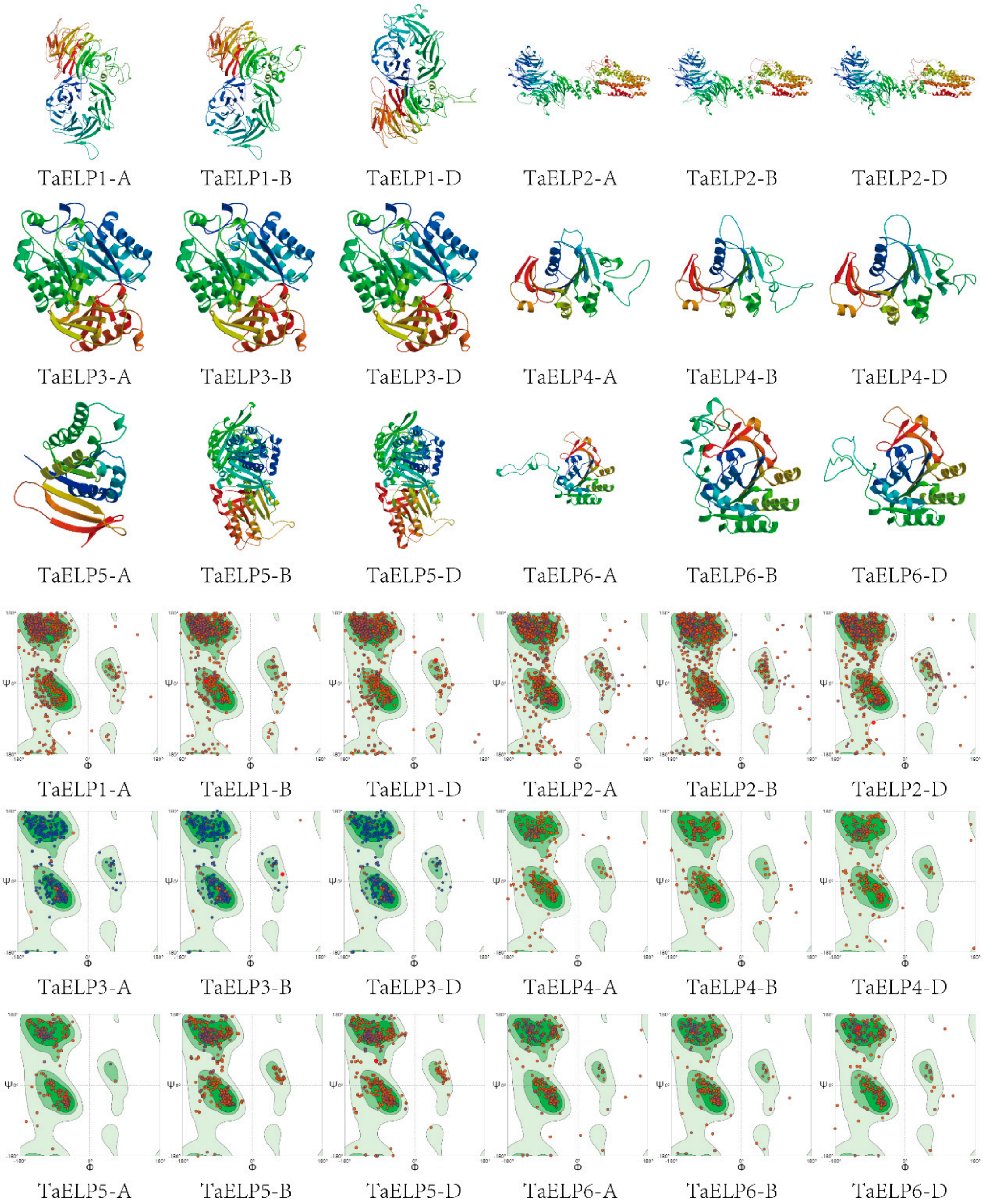

Figure S1. Schematic illustration of 3-D protein structures and the Ramachandran plot for TaELP proteins.

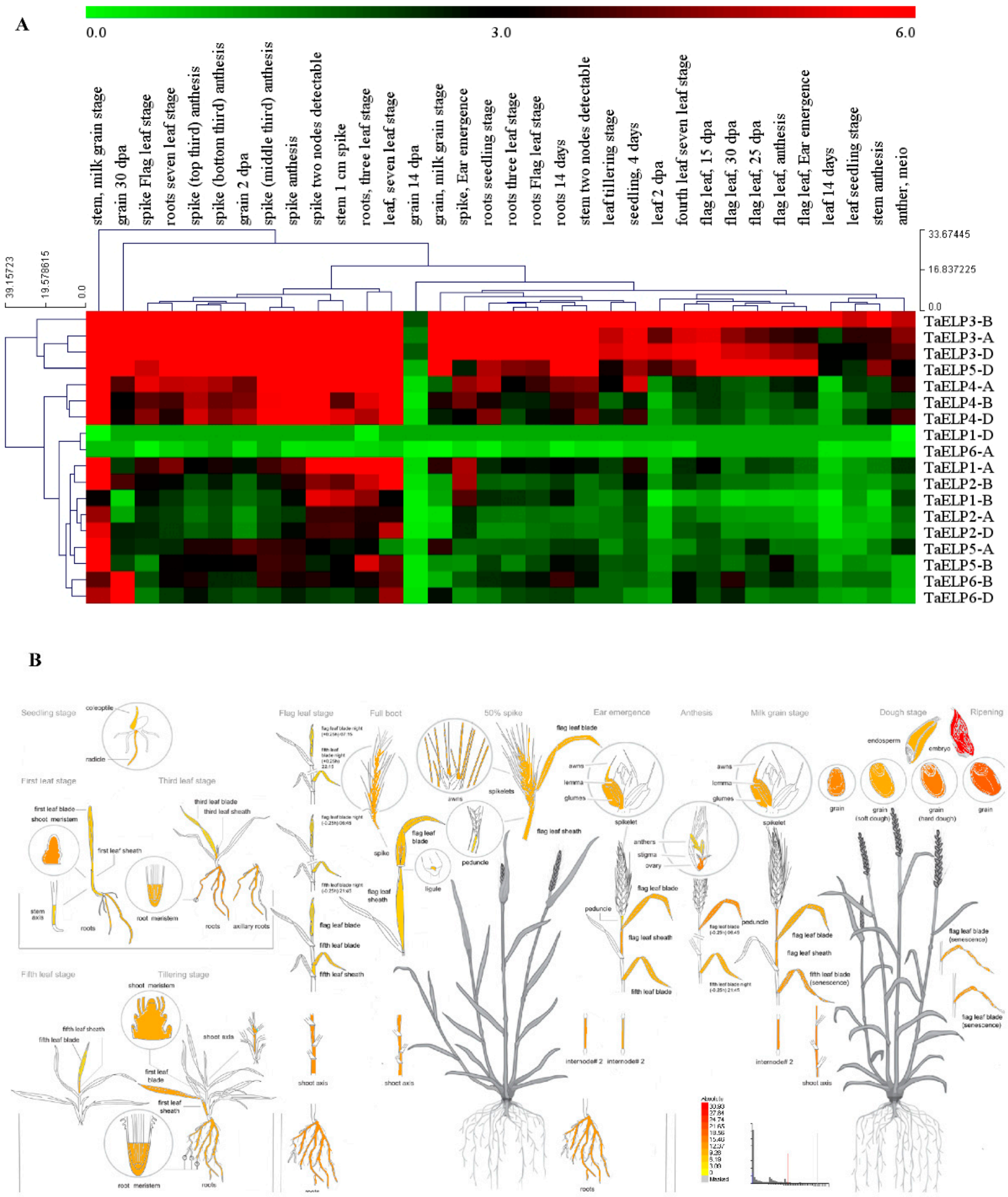

Figure S2. (A) Heatmap representing the expression pattern of *TaELP* genes in different organs and developmental stages. (B) The expression of the *TaELP3* gene in different organs of wheat predicted by Wheat eFP Browser.

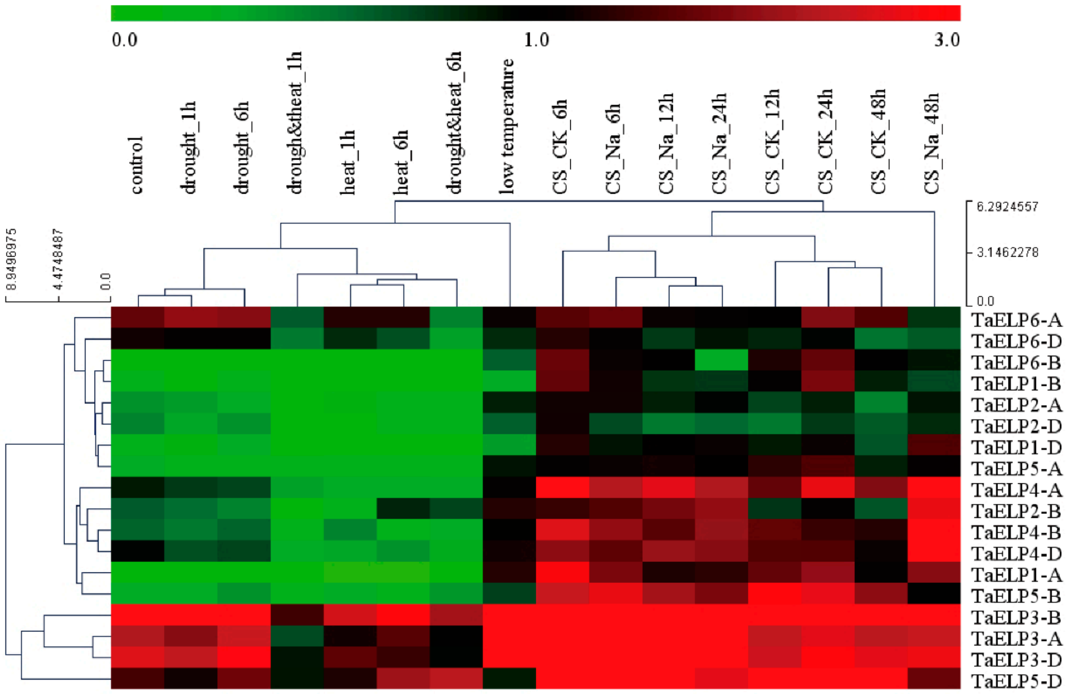

Figure S3: Heatmap representing the expression patterns of *TaELP* genes under different abiotic stress conditions. The TPM values are used directly to build the heatmap. CS: Chinese Spring Na: salt stress h: hours.

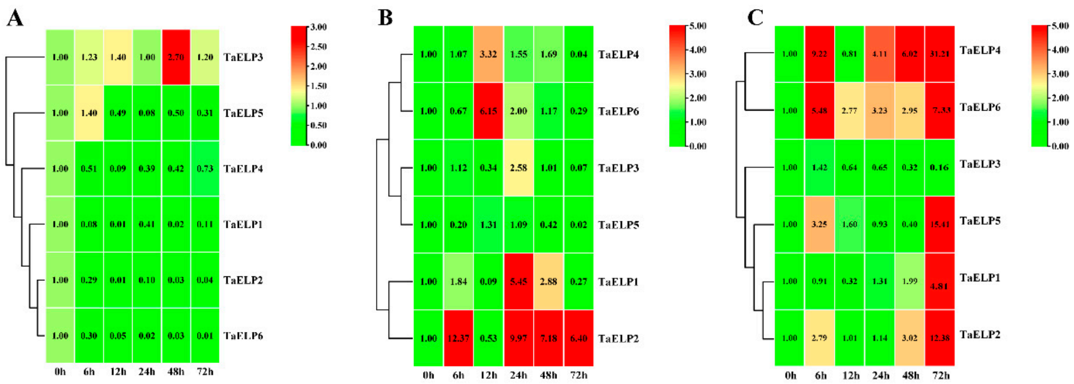

Figure S4. Relative transcript profiles of *TaELPs* in response to (A) Indole-3-acetic acid (IAA), (B) salicylic acids (SA), (C) abscisic acid (ABA). The relative transcripts of all genes were analyzed using qRT-PCR. The relative transcript levels of *TaELPs* were measured using the comparative threshold ($2^{-\Delta\Delta CT}$) method. Data normalized with the transcripts of wheat elongation factor, *TaEF-1α*. The 0 h post-treatment (A, B, C) was used as a control and standardized with 1. Red and green colors denote strong and weak transcription of *TaELPs*, respectively. The heat map was generated with TBtools and the tree was constructed with the average linkage clustering method.

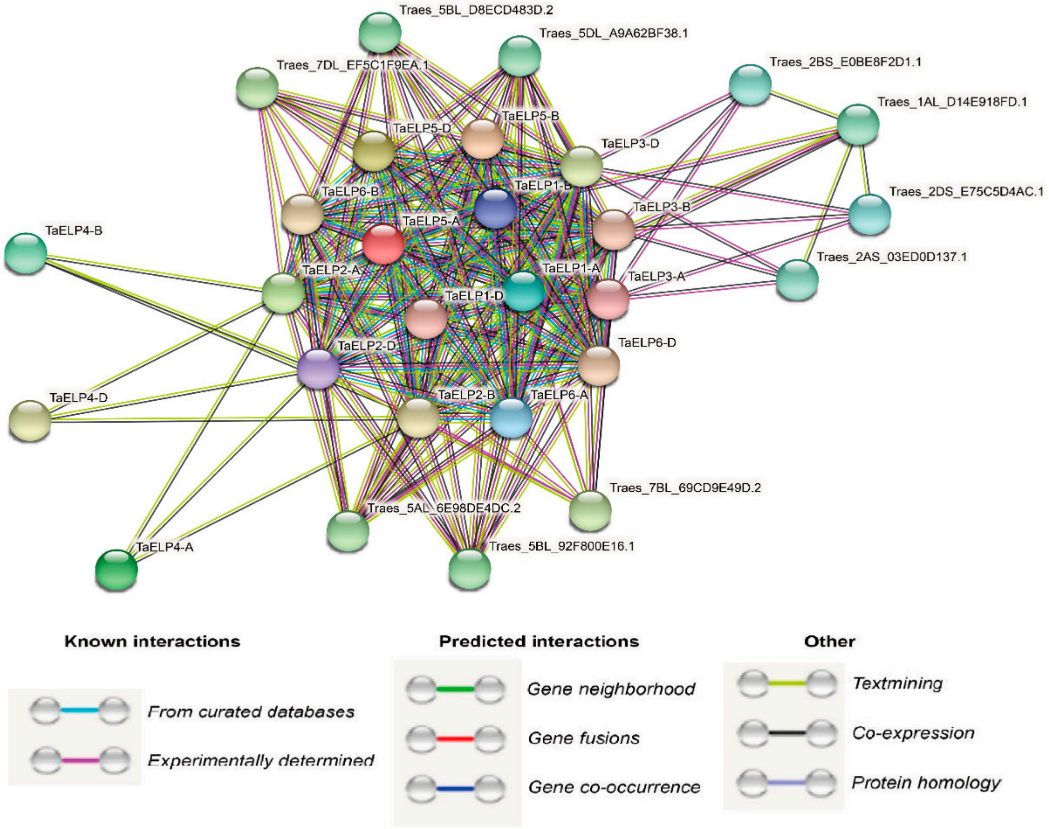

Figure S5. Protein-protein interaction analysis of TaELP proteins. Protein-protein interaction network generated by STRINGV9.1. Each node represents a protein and each edge represents an interaction, colored by evidence type.
